# Acute Recreational Noise-Induced Cochlear Synaptic Dysfunction in Humans With Normal Hearing: A Prospective Cohort Study

**DOI:** 10.3389/fnins.2021.659011

**Published:** 2021-04-09

**Authors:** Qixuan Wang, Lu Yang, Minfei Qian, Yingying Hong, Xueling Wang, Zhiwu Huang, Hao Wu

**Affiliations:** ^1^Department of Otolaryngology-Head and Neck Surgery, Ninth People’s Hospital, Shanghai Jiao Tong University School of Medicine, Shanghai, China; ^2^Ear Institute, Shanghai Jiao Tong University School of Medicine, Shanghai, China; ^3^Shanghai Key Laboratory of Translational Medicine on Ear and Nose Diseases, Shanghai, China; ^4^Hearing and Speech Center, Ninth People’s Hospital, Shanghai Jiao Tong University School of Medicine, Shanghai, China; ^5^Biobank, Ninth People’s Hospital, Shanghai Jiao Tong University School of Medicine, Shanghai, China

**Keywords:** noise-induced hearing loss, acute recreational noise exposure, hidden hearing loss, cochlear synaptopathy, auditory brainstem response, speech recognition in noise

## Abstract

**Objectives:**

The objective of the study was to identify the acute high-intensity recreational noise-induced effects on auditory function, especially the cochlear synaptopathy-related audiological metrics, in humans with normal hearing.

**Methods:**

This prospective cohort study enrolled 32 young adults (14 males and 18 females); the mean age was 24.1 ± 2.4 years (ranging from 20 to 29). All participants with normal hearing (audiometric thresholds ≤25 dB HL at frequencies of 0.25, 0.5, 1, 2, 3, 4, 6, and 8 kHz for both ears) had already decided to participate in the outdoor music festival. Participants were asked to measure the noise exposure dose and complete auditory examinations, including the air-conduction pure-tone audiometry (PTA), distortion product otoacoustic emission (DPOAE), contralateral suppression (CS) on transient evoked otoacoustic emission (TEOAE), auditory brainstem response (ABR) test and Mandarin Hearing in Noise Test (MHINT), at baseline and 1 day and 14 days after music festival noise exposure.

**Results:**

The mean time of attending the music festival was 7.34 ± 0.63 h (ranging from 6.4 to 9.5), the mean time-weighted average (TWA) of noise exposure dose was 93.2 ± 2.39 dB(A) (ranging from 87.9 to 97.7). At neither 1 day nor 14 days post exposure, there were no statistically significant effects on PTA thresholds, DPOAE amplitudes, CS on TEOAEs, or MHINT signal-to-noise ratios (SNRs) of acute outdoor music festival noise exposure, regardless of sex. While the ABR wave I amplitudes significantly decreased at 1 day after exposure and recovered at 14 days after exposure, the exposed/unexposed ABR wave I amplitude ratio was significantly correlated with MHINT SNR change at 1 day after exposure, although it was not correlated with the noise exposure dose.

**Conclusion:**

In young adults with normal hearing, we found the self-compared decrement of ABR wave I amplitudes at 1 day post acute recreational noise exposure at high intensity, which also contributes to the change in speech perceptual ability in noisy backgrounds. This study indicated that auditory electrophysiological metric changes might be a more sensitive and efficient indicator of noise-induced cochlear synaptic dysfunction in humans. More attention should be paid to the recreational noise-induced cochlear synaptopathy and auditory perceptual disorder.

## Introduction

According to the United States Centers for Disease Control and Prevention, 14% of adults aged 20–69 years have hearing loss (Hoffman et al., 2017). Noise exposure is the most common environmental factor causing hearing loss in adults; noise-induced hearing loss (NIHL) may occur even due to daily noise exposure, such as loud music at concerts (Eichwald et al., 2018), portable media players and earphones (le Clercq et al., 2018), and public transport (Yao et al., 2017). There have been many concerns about the trend of increasing incidence of NIHL since noise exposure is unexpectedly pervasive in modern life, especially in younger populations (Murphy et al., 2018).

A recent mouse study demonstrated that even moderate noise exposure that induces a “temporary” hearing threshold shift (TTS) could result in permanent loss of ribbon synapses accompanied with abnormal suprathreshold auditory brainstem response (ABR), which was known as the cochlear synaptopathy (Kujawa and Liberman, 2009). Several studies further indicated that the cochlear synaptopathy might be the primary cause of hearing difficulties in individuals with normal hearing thresholds, which has been referred to as “hidden hearing loss” (HHL) (Kujawa and Liberman, 2009; Mehraei et al., 2016; Lobarinas et al., 2017). Recent surveys reported that approximately 12–15% of the population with normal hearing thresholds might have the HHL (Kohrman et al., 2020), which also contribute to tinnitus (Schaette and McAlpine, 2011; Gu et al., 2012) and age-related hearing loss (Sergeyenko et al., 2013; Fernandez et al., 2015; Liberman et al., 2015). However, it remains unknown whether daily loud recreational noise exposure could induce the irreversible HHL. Although many studies have made efforts in the identification of the noise-induced HHL-related auditory function changes in humans, this topic remains controversial, mainly due to the difficulty in controlling of the noise exposure and self-comparison data before and after noise exposure.

Most noise-induced HHL studies are based on retrospective design, and conclusions of which are inconsistent. A number of studies have suggested that individuals with experiences of loud noise exposure have greater difficulties in complex listening tasks under noisy background environments (Liberman et al., 2016) and decreased suprathreshold stimulating peak I amplitudes of ABR and electrocochleogram (Stamper and Johnson, 2015; Liberman et al., 2016), despite the normal audiological thresholds. In contrast, some studies failed to associate the noise exposure experience with audiological electrophysiology or perception measures in humans (Prendergast et al., 2017a,b).

To date, there are still very few prospective studies on recreational noise-induced HHL. The only self-comparison evidence from 26 young adults with normal hearing found no permanent auditory function changes after the recreational noise exposure, suggesting little risk of HHL (Grinn et al., 2017). However, in that study, recreational events included movie, bar music, concert, and dance at noise exposure level of mean 92.7 ± 7.7 dB(A) (ranging from 73.1 to 104.2) for 3.3 ± 0.9 h (ranging 1.5–4.5 h). It is still unknown whether louder recreational events would cause the cochlear synaptopathy or HHL in consistent with animal studies.

Outdoor music festivals, which include multiple concerts and often last for several hours, have recently become increasingly popular and should be a considerable source of recreational noise exposure. A recent study including 51 young adults observed the TTS after an outdoor music festival lasting 4.5 h at approximately 100 dB(A) noise exposure (Kraaijenga et al., 2018). Here, we conducted a prospective cohort study including 32 normal-hearing young adults who participated in the outdoor music festival with personal sound level measurements, in order to identify whether the acute recreational noise exposure at high intensity would contribute to cochlear synaptopathy or auditory perceptual disorder.

## Materials and Methods

### Subjects

We recruited volunteers from young adults who had already decided to participate in the outdoor music festival in eastern China. There were 47 healthy participants aged 20–29 years initially recruited, and 32 (14 males and 18 females, sex was self-reported) with normal hearing were included based on the following criteria: (1) no family history of hearing loss, no history of otological injuries or diseases, no history of occupational noise exposure, and no history of outdoor music festival noise exposure within 2 months before participation in this study; (2) both ears show normal external ear canal and tympanic membrane with otoscope, type A tympanogram with 226 Hz probe tone, and air-conduction pure tone audiometric thresholds ≤25 dB HL at frequencies of 0.25, 0.5, 1, 2, 3, 4, 6, and 8 kHz; and (3) have enough language ability for the mandarin Chinese speech recognition test.

### Procedure

Subjects were requested to complete the basic information collection (including age, previous visits of music festival/concert/night club, earphone use, and self-reported hearing difficulty) and baseline auditory function examinations within 1 week before participation in the outdoor music festival. Participants with self-reported tinnitus were asked to complete the mandarin Tinnitus Handicap Inventory (THI) (Meng et al., 2012). The noise exposure dose during the festival was measured for each subject. Follow-up auditory function examinations were performed at 1 day post exposure (follow-up 1) and 14 days post exposure (follow-up 2) of the outdoor music festival. The noise exposure dose received by each subject during the outdoor music festival was measured using a personal sound exposure meter (ASV 5910 type, Hangzhou Aihua, China). Subjects were requested to wear the instrument on their shoulders (near the auricle level), and the duration of festival visits, time-weighted average (TWA), and the C-weighted peak level (*L*_*Cpeak*_) of the noise exposure from the beginning to the end of attendance of the music festival were recorded.

**FIGURE 1:**
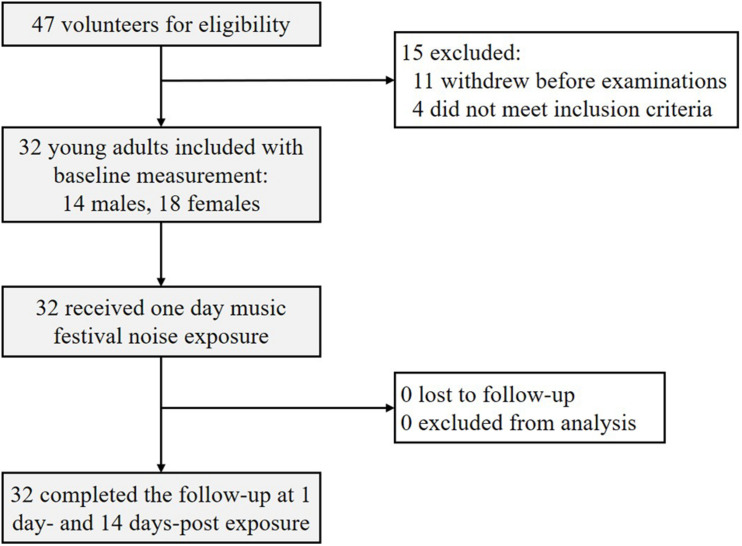
Flowchart of this study. Among 47 initial volunteers, 32 young participants with normal hearing were enrolled and completed this two-stage follow-up study.

Figure 1 shows the flowchart of this cohort study. All protocols and procedures were approved by the ethics committee of the Ninth People’s Hospital affiliated with Shanghai Jiao Tong University School of Medicine. All participants signed written informed consent forms and were informed that participation can be withdrawn at any time. Subjects were offered a 600 (China Yuan) stipend after completion of the study.

### Auditory Function Examinations

Auditory function examinations at unexposed baseline and follow-up included the air-conduction pure-tone audiometry (PTA), distortion product otoacoustic emissions (DPOAEs), and contralateral suppression (CS) on transient evoked otoacoustic emissions (TEOAEs), the ABR test and the Mandarin Hearing in Noise Test (MHINT), which were performed by certified audiological technicians in a soundproof and electromagnetic shielding booth [background noise level <25 dB(A)].

Pure-tone audiometry at frequencies of 0.25, 0.5, 1, 2, 3, 4, 6, and 8 kHz in both ears was performed using an audiometer (Madsen Astera, GN Otometrics, Denmark) with inserted earphones in accordance with the regulations of ISO 8253-1:2010.

Distortion product otoacoustic emission tests were performed using a cochlear emission analyzer (Capella, GN Otometrics, Denmark), which were considered valid when the emission amplitude exceeded the noise by at least 3 dB. DPOAEs were elicited by two tones [L_1_ = 65 dB sound pressure level (SPL), L_2_ = 55 dB SPL]; the determined f_2_/f_1_ ratio was equal to 1.22, and the 2f_1_–f_2_ cubic distortion product (DP1) component for each pair of stimuli was recorded at frequencies of 1, 2, 3, 4, 6, and 8 kHz. The probe fitting check and the two-tone adjustments were performed before each measurement session.

Contralateral suppression on TEOAEs is a reliable measure to monitor the medial olivocochlear (MOC) efferent reflex status over time (Stuart and Cobb, 2015). In this study, TEOAEs were evoked with 60 dB peak equivalent SPL (peSPL) linear click stimuli at a rate of 19.3/s with and without a contralateral 50 dB SL white noise suppressor (delivered by the audiometer and insert earphone) without probe removal. The intensity of this suppressor stimulus was well below the threshold of the stapedial muscle reflex for all the subjects. Responses were averaged to 2,080 sweeps, and the stimulus stability was at least 90%. Suppression was calculated by subtracting the TEOAE amplitude with contralateral stimulation from those without contralateral stimulation. The frequency bands measured were centered at frequencies of 1, 2, 3, 4, and 5 kHz, and all frequencies were averaged.

Auditory brainstem response tests were performed using SmartEP (InteIIegent Hearing System, United States). The recording electrode was placed on the high forehead, the reference electrode was placed on the mastoid, and the grounding electrode was placed on the low forehead. Electrode impedance was less than 5 kΩ. Stimuli were presented using 100-μs clicks at 90 dB normal hearing level (nHL) with alternating polarity at a rate of 11.1/s via insert earphones (ER-3C, Etymotic Research, United States). Waveforms were collected, passed through a bandpass filter from 100 to 3,000 Hz, and averaged across 1,024 stimulus presentations. Two replications of each waveform were obtained, and the peak amplitude of wave I was calculated according to a previous study (Stamper and Johnson, 2015).

We used MHINT (Wong et al., 2007) consisting of 12 lists, each containing 20 sentences, and each sentence contained 10 Chinese characters. The BLIMP software (version 1.3, House Ear Institute, United States) was used to present the sentences at various signal-to-noise ratio (SNR) controls via a personal computer and headphones (HD200, Sennheiser, Germany). The test followed an adaptive procedure as previously described (Zhang et al., 2010). During the test, the ipsilateral white noise level was fixed at 65 dB(A), and the first sentence was presented at 0 dB SNR; the conventional rule required that the entire sentence be repeated accurately. SNR was finally calculated at the presentation level necessary for a listener to recognize the sentence materials correctly 50% of the time.

### Statistical Analysis

Data analysis was performed using IBM SPSS software (version 24.0, SPSS Inc., United States) and Prism (version 8.0, GraphPad Software, United States). Continuous variables are presented as the mean ± SD, and categorical variables are presented as percentages [*n* (%)]. The normality of continuous variables was assessed by the Kolmogorov–Smirnov test. Characteristics, MHINT SNRs and ABR wave I amplitudes at baseline between males and females were compared using unpaired *t*-tests or χ^2^ tests. Differences in PTA thresholds, DPOAE amplitudes, and CS on TEOAEs between males and females were analyzed using the two-way repeated measures ANOVA with sexes and frequencies as dependent variables. Differences in PTA thresholds, DPOAE amplitudes, and CS on TEOAEs between the baseline, follow-up 1, and follow-up 2 groups were analyzed using the two-way repeated measures ANOVA with noise exposure and frequencies as dependent variables. MHINT SNRs and ABR wave I amplitudes between baseline, follow-up 1, and follow-up 2 groups were analyzed using the two-way repeated measures ANOVA with noise exposure and sexes as dependent variables. Pearson correlation analysis was used to screen the significant association between the TWA and auditory function changes; then correlations of the TWA, exposed/unexposed ABR wave I amplitudes ratio, and MHINT SNR changes were determined using linear regression analysis. A two-tailed *P* < 0.05 was considered statistically significant.

## Results

### Characteristics of Subjects

A total of 32 participants aged 20–29 years [14 males (43.8%), 18 females (56.2%), mean age: 24.1 ± 2.4 years] who completed this study were included in the analyses. The distributions of characteristics at baseline (age, previous visits of music festival/concert/night club, earphone use, and self-reported hearing difficulty) and during the outdoor music festival (duration of festival visit and TWA of noise exposure) were not significantly different between males and females, while the mean *L*_*Cpeak*_ of females was slightly higher than that of males (shown in Table 1). We noticed that nine participants (three males and six females) experienced at least one self-reported tinnitus, but none of them was indeed troubled from tinnitus according to THI scores. Among all the participants, the mean duration of festival visits was 7.34 ± 0.63 h (ranging from 6.4 to 9.5), the mean TWA was 93.2 ± 2.39 dB(A) (ranging from 87.9 to 97.7), and the mean *L*_*Cpeak*_ was 135.4 ± 4.1 dB (ranging from 129.9 to 139.6). None of the participants used hearing protective devices such as earplugs during the festival visit.

**TABLE 1:** Participant characteristics at baseline and during the music festival.

Characteristics	Males (*n* = 14)	Females (*n* = 18)	*t/*χ*^2^*	*P*
Age, mean (SD)	25 (2.9)	23 (1.7)	1.888	0.069
Previous visits to music festivals/concerts/nightclubs			4.092	0.129
≤once/year, *n* (%)	5 (35.7)	2 (11.1)		
≥2 times/year, *n* (%)	7 (50)	15 (83.3)		
≥2 times/month, *n* (%)	2 (14.3)	1 (5.6)		
Tinnitus history			0.552	0.759
Almost never, *n* (%)	11 (78.6)	12 (66.7)		
Yes, spontaneously, *n* (%)	2 (14.3)	4 (22.2)		
Yes, after exposure to noise, *n* (%)	1 (7.1)	2 (11.1)		
Previous use of personal earphones			1.169	0.557
Almost never, *n* (%)	2 (14.3)	5 (27.8)		
0–2 h/day, *n* (%)	8 (57.1)	10 (55.6)		
2–5 h/day, *n* (%)	4 (28.6)	3 (16.7)		
Previous use of earplugs			0.803	0.370
Almost never, *n* (%)	14 (100)	17 (94.4)		
Yes, *n* (%)	0 (0)	1 (5.6)		
Self-reported hearing difficulty			3.418	0.181
Almost never, *n* (%)	5 (35.7)	2 (11.1)		
Yes, only amid noise, *n* (%)	6 (42.9)	13 (72.2)		
Yes, in daily life, *n* (%)	3 (21.3)	3 (16.7)		
During festival				
Duration of visit, mean (SD), hours	7.2 (0.9)	7.4 (0.4)	–0.750	0.463
TWA, mean (SD), dB(A)	93.8 (1.6)	93.4 (1.8)	0.769	0.448
*L*_*Cpeak*_, mean (SD), dB	136.7 (4.0)	133.8 (3.7)	–2.074	0.047

*SD, standard deviation; TWA, time-weighted average. There were no statistically significant differences in most characteristics except for the L_Cpeak_ between male and female participants. Analyses were performed by the unpaired t-test or χ^2^ test.*

**FIGURE 2:**
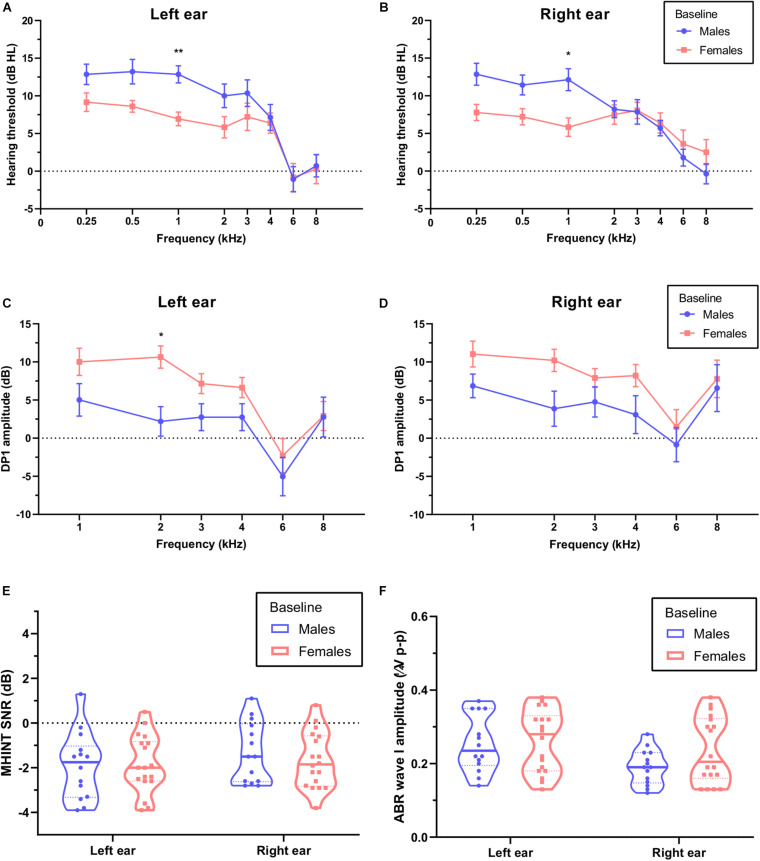
Comparison of auditory function between males and females at baseline. Females (pink) show a lower pure-tone audiometry (PTA) threshold at a 1 kHz frequency than males (blue) for the left ear **(A)** and the right ear **(B)**. Females show higher distortion product otoacoustic emission (DPOAE) amplitudes at a 1 kHz frequency than males for the left ear **(C)** but not the right ear **(D)**, analyses were performed by the two-way repeated measures ANOVA. There were no statistically significant differences in signal-to-noise ratios (SNRs) of Mandarin Hearing in Noise Test (MHINT) **(E)** and auditory brainstem response (ABR) wave I amplitudes **(F)** between males and females for either ear, analyses were performed by the unpaired *t*-test. *P < 0.05, **P < 0.01.

Although all the participants showed normal hearing at baseline with PTA thresholds ≤25 dB HL, females showed a lower PTA threshold at a 1-kHz frequency for both the left ear (Figure 2A, *P* = 0.003) and the right ear (Figure 2B, *P* = 0.021) and a higher DPOAE DP1 amplitude at a 2-kHz frequency for the left ear (Figure 2D, *P* = 0.011) than males. We did not observe significant differences in DPOAE DP1 amplitudes for the right ear (*P* = 0.058), CS on TEOAEs (see Table 2) for both left (*P* = 0.841) and right (*P* = 0.608) ears, MHINT SNRs (see Figure 2E) for both left (*P* = 0.999) and right (*P* = 0.916) ears, or ABR wave I peak amplitudes (see Figure 2F) for both left (*P* = 0.999) and right (*P* = 0.197) ears between males and females.

**TABLE 2:** Effects of noise exposure on contralateral suppression (CS) on transient evoked otoacoustic emissions (TEOAEs).

CS on TEOAEs (dB)	Males (*n* = 14)						Females (*n* = 18)					
	Left ear			Right ear			Left ear			Right ear		
	Before	1 day post	14 days post	Before	1 day post	14 days post	Before	1 day post	14 days post	Before	1 day post	14 days post
1 kHz	1.10 (3.22)	1.84 (2.26)	1.11 (3.41)	2.09 (1.99)	2.22 (2.27)	2.31 (2.54)	2.43 (2.36)	1.35 (2.79)	2.06 (2.66)	2.82 (2.74)	2.00 (2.29)	2.38 (2.19)
2 kHz	1.21 (3.22)	1.33 (2.76)	1.68 (2.97)	1.41 (2.28)	1.52 (1.57)	2.15 (2.43)	2.57 (2.75)	1.85 (2.16)	2.03 (2.87)	2.99 (3.14)	2.37 (2.26)	2.67 (3.04)
3 kHz	1.86 (2.33)	1.34 (1.74)	1.14 (2.58)	1.40 (2.49)	1.24 (3.28)	1.11 (1.98)	1.68 (3.02)	0.73 (1.79)	1.57 (1.77)	2.26 (2.55)	2.24 (2.48)	2.68 (2.10)
4 kHz	1.21 (2.56)	1.64 (2.81)	1.19 (2.14)	1.01 (2.15)	1.11 (1.76)	1.85 (1.65)	1.73 (1.33)	1.10 (1.70)	1.43 (1.45)	1.38 (2.16)	1.13 (1.81)	1.24 (1.53)
5 kHz	1.15 (2.18)	0.87 (1.73)	1.04 (1.99)	0.54 (1.29)	1.00 (1.71)	0.14 (1.6)	0.64 (2.16)	0.74 (1.86)	0.85 (2.05)	1.11 (2.81)	0.80 (2.78)	1.47 (1.85)
All	1.99 (2.31)	1.91 (1.69)	1.63 (2.69)	1.86 (1.82)	1.91 (0.95)	2.05 (1.49)	2.16 (1.97)	1.34 (1.79)	2.24 (1.86)	2.45 (2.49)	1.82 (1.95)	2.77 (1.65)

*There are no statistically significant differences in CS on TEOAEs over 1–5 kHz frequencies and average value between males and females at baseline (before noise exposure) for the left ear (P = 0.841) and right ear (P = 0.608). There are no statistically significant differences in CS on TEOAEs over 1–5 kHz frequencies and average value of males (P = 0.248 for left ear, P = 0.161 for right ear) or females (P = 0.333 for left ear, P = 0.576 for left ear) between the baseline, 1 day and 14 days post the outdoor music festival noise exposure. Analyses were performed by the two-way repeated measures ANOVA.*

### Auditory Function Changes Caused by Outdoor Music Festivals

**FIGURE 3:**
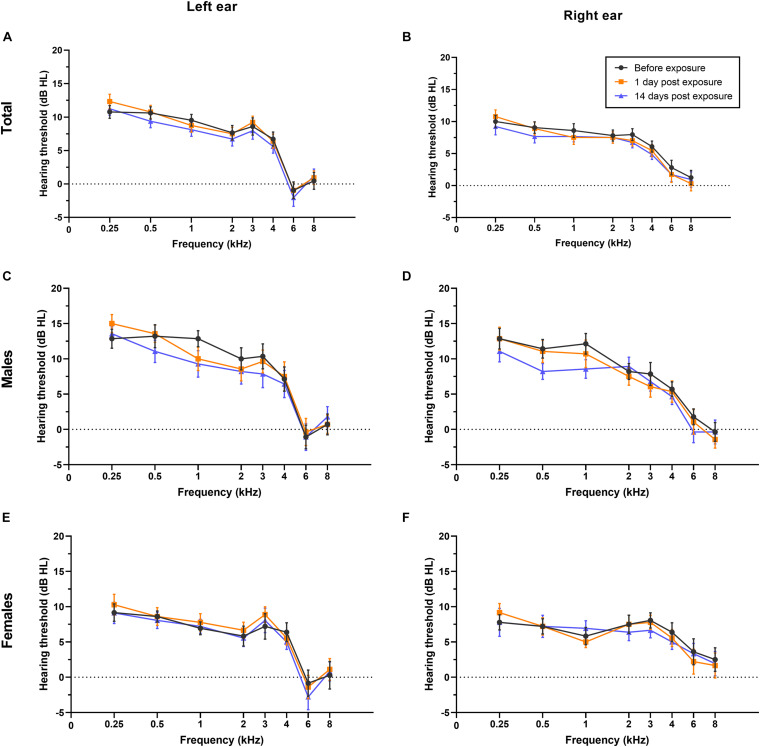
Effects of noise exposure on PTA. There were no statistically significant differences in the PTA hearing thresholds of either ear at frequencies of 0.25–8 kHz in the overall sample **(A,B)**, in males specifically **(C,D)**, or in females specifically **(E,F)** among baseline (black), 1 day after outdoor music festival noise exposure (orange) and 14 days (blue) after exposure. Analyses were performed by the two-way repeated measures ANOVA.

**FIGURE 4:**
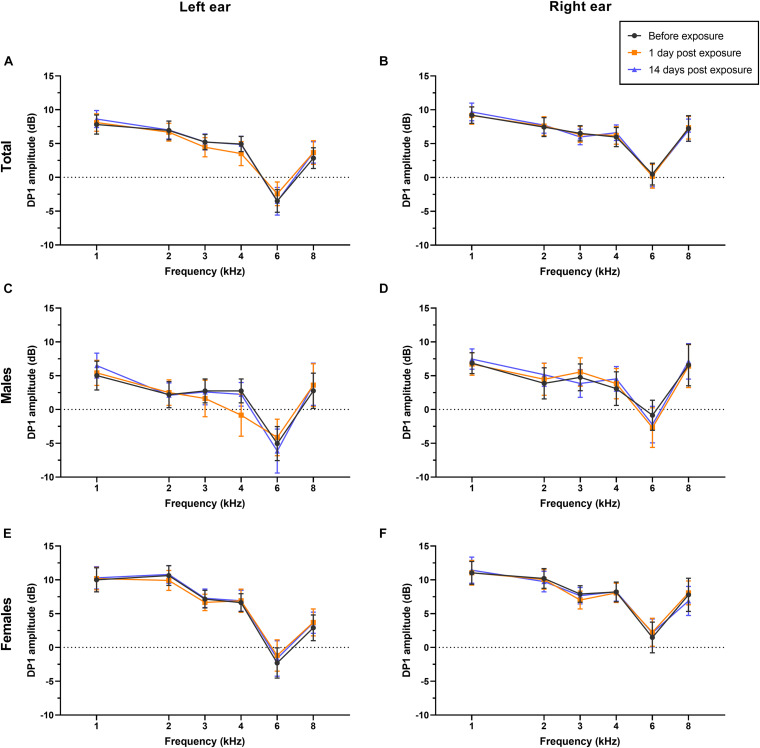
Effects of noise exposure on DPOAE. There were no statistically significant differences in the DPOAE amplitudes of either ear at frequencies of 1–8 kHz in the overall sample **(A,B)**, in males specifically **(C,D)**, or in females specifically **(E,F)** among baseline (black), 1 day after outdoor music festival noise exposure (orange) and 14 days (blue) after exposure. Analyses were performed by the two-way repeated measures ANOVA.

**FIGURE 5:**
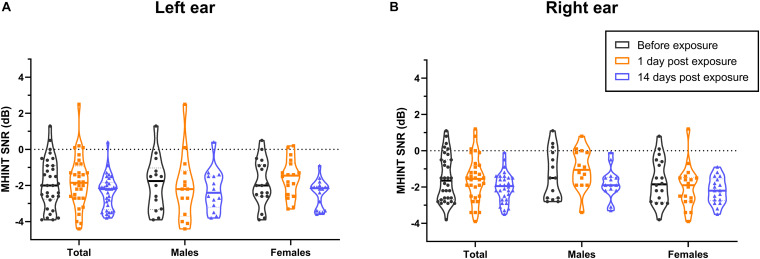
Effects of noise exposure on MHINT. There were no statistically significant differences in the SNRs of MHINT in the overall sample, in males specifically, or in females specifically among baseline (black), 1 day after outdoor music festival noise exposure (orange) and 14 days (blue) after exposure for the left ear **(A)** or the right ear **(B)**. Analyses were performed by the two-way repeated measures ANOVA.

Overall, in this study, PTA thresholds (total: *P* = 0.699 for left ear, *P* = 0.591 for right ear; males: *P* = 0.775 for left ear, *P* = 0.509 for right ear; females: *P* = 0.816 for left ear, *P* = 0.931 for right ear; see Figure 3), DPOAE DP1 amplitudes (total: *P* = 0.955 for left ear, *P* = 0.997 for right ear; males: *P* = 0.947 for left ear, *P* = 0.984 for right ear; females: *P* = 0.974 for left ear, *P* = 0.997 for right ear; see Figure 4), CS on TEOAEs (total: *P* = 0.936 for left ear, *P* = 0.560 for right ear; males: *P* = 0.248 for left ear, *P* = 0.161 for right ear; females: *P* = 0.333 for left ear, *P* = 0.576 for right ear; see Table 2), and MHINT SNRs (total: *P* = 0.999 for left ear, *P* = 0.999 for right ear; males: *P* = 0.999 for left ear, *P* = 0.583 for right ear; females: *P* = 0.859 for left ear, *P* = 0.598 for right ear; see Figure 5) at 1 day or 14 days after the outdoor music festival noise exposure were comparable with those at unexposed baseline for both ears, despite sex.

**FIGURE 6:**
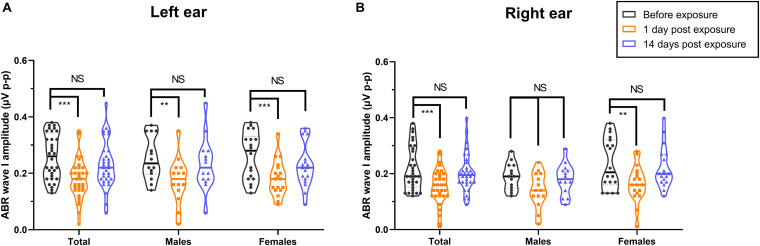
Effects of noise exposure on ABR wave I amplitudes. The peak amplitude of wave I significantly decreased at 1 day after outdoor music festival noise exposure (orange) and recovered to the baseline level (black) at 14 days after exposure (blue) in the left **(A)** and right **(B)** ears of total participants and of females specifically, as well as in the left ear of males, but not in the right ear of males. Analyses were performed by the two-way repeated measures ANOVA. NS, no significance. **P < 0.01, ***P < 0.001.

Notably, we observed that the peak amplitudes of ABR wave I significantly decreased at 1 day after exposure (Figure 6A for left ear, Figure 6B for right ear), with a recovery at 14 days after exposure among all the participants (both *P*-values < 0.001). For females, the peak amplitudes at 1 day after exposure were significantly lower than baseline for both the left ear (*P* < 0.001) and the right ear (*P* = 0.008). For males, the decrement of peak amplitudes 1 day after exposure was significantly different from baseline only for the left ear (*P* = 0.002) but not the right ear (*P* = 0.055). The mean ABR waveforms are shown in Supplementary Figure 1.

### Relationship Between Noise Exposure Dose and Auditory Function

**FIGURE 7:**
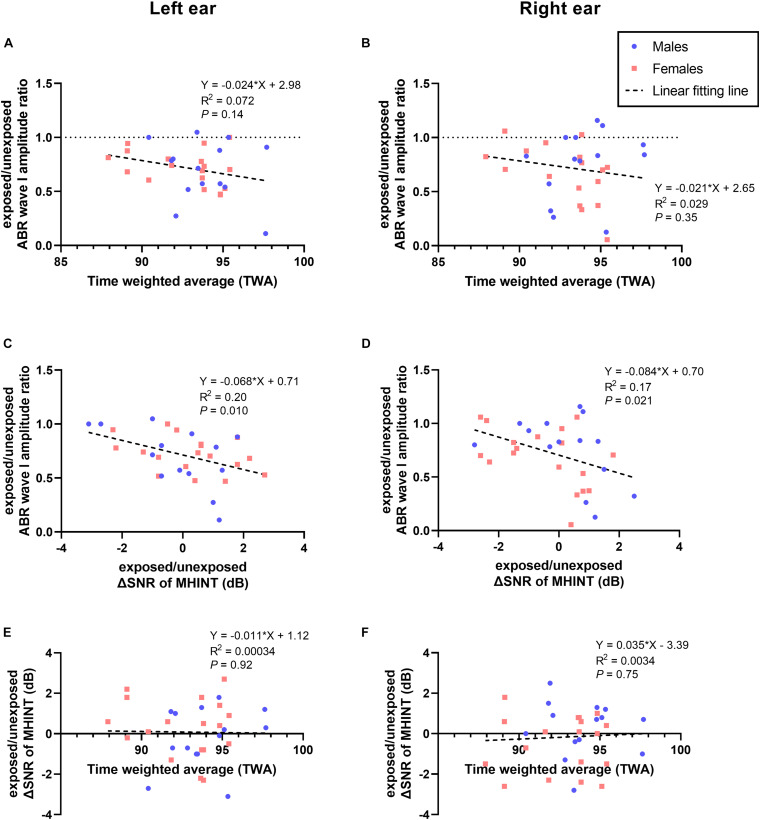
Correlations of noise exposure, ABR wave I, and MHINT. Neither the exposed/unexposed ABR wave I amplitude ratio **(A,B)** nor the SNR changes (ΔSNR) of the MHINT **(E,F)** significantly correlated with time-weighted average (TWA) noise exposure, whereas the exposed/unexposed ABR wave I amplitude ratio significantly correlated with the ΔSNR of MHINT **(C,D)** for the left ear and right ear. Males are in blue, females are in pink, and lines of fit for the overall sample are in black.

To further explore the association between the acute outdoor music festival noise exposure dose and auditory function changes, we performed Pearson correlation analysis but did not observe any statistically significant correlation (see Supplementary Figure 2). However, only the normalized exposed/unexposed ABR wave I amplitude changes (ratio of amplitude at 1 day post exposure to amplitude at baseline) seems to show a decreasing tendency with higher noise exposure doses for both ears (Figure 7A for the left ear, *P* = 0.14; Figure 7B for the right ear, *P* = 0.35). Correlation analyses were also performed to explore the relationship between several auditory function changes in this study. We found that the exposed/unexposed ABR wave I amplitude ratio was significantly associated with MHINT SNR changes at 1 day after the outdoor music festival noise exposure (see Figure 7C for the left ear, *P* = 0.010; Figure 7D for the right ear, *P* = 0.021), although it was not significantly correlated with the noise exposure dose (see Figure 7E for the left ear, *P* = 0.92; Figure 7F for the right ear, *P* = 0.75).

## Discussion

In this study, to identify whether acute high-level recreational noise exposure would induce HHL or other audiological impairments in humans, we followed up on the temporary and sustained changes in auditory function in 32 normal-hearing young adults who attended the outdoor music festival. To our knowledge, this is the first prospective cohort study that identified that the auditory electrophysiological indicator of suprathreshold stimulating ABR wave I amplitude decreased transiently with subsequent recovery after acute loud recreational noise exposure, without other significant auditory functional changes in humans. In addition, we found that the ABR wave I amplitude changed in relation to speech recognition ability in noisy environments after noise exposure, although the correlations between auditory function changes and noise exposure dose were not significant.

In consideration of the sex differences in auditory characteristics and susceptibility to NIHL (Wang et al., 2021), we compared auditory function between males and females at baseline. Generally, consistent with previous studies (Delhez et al., 2020), females showed a slightly better PTA threshold and DPOAE amplitude (see Figure 2) than males at baseline, which would not affect the efficiency of conclusion in this study, since we analyzed the auditory function changes not only in participants overall but also in males and females separately.

### Acute Recreational Noise-Induced Auditory Effects Depend on Exposure Doses

Numerous recent studies have indicated that long-term exposure to high-intensity recreational and professional music potentially increases the risk of hearing loss (Schink et al., 2014; Pouryaghoub et al., 2017; le Clercq et al., 2018). Several previous studies that had well-quantified exposure doses demonstrated that short-term music exposure at high intensity has the potential to induce a TTS. According to a randomized clinical trial in Amsterdam to assess the effectiveness of earplugs in preventing TTSs following music exposure, 22 of 52 ears (42%) among the normal-hearing adult volunteers who experienced unprotected outdoor music exposure [TWA approximately 100 dB(A) during the festival] showed a TTS over frequencies of 3 and 4 kHz and a significant decrement in DPOAE amplitude over frequencies of 2–8 kHz (Ramakers et al., 2016). Le Prell et al. (2012) described the effects of carefully controlled 4-h digital audio player use for three different music listening levels [at 93–95, 98–100, and 100–102 dB(A)] on audiometric threshold changes of 33 normal-hearing young adult college students. The largest TTS was observed at a 4-kHz frequency (averaged 6.3 ± 3.9 dB, ranging from 0 to 13 dB) 15 min after higher levels of sound exposure, which almost recovered completely within the first 4 h after exposure. In contrast, in another study on young participants with a normal hearing threshold, there were no statistically significant correlations between noise exposure and changes in audiometric threshold or DPOAE amplitude either the day after the loud event [based on noise exposure level of 92.7 ± 7.7 dB(A), range 73.1−104.2 dB(A) for 3.3 ± 0.9 h (range 1.5−4.5 h)] or 1 week later (Grinn et al., 2017).

In our study, the sound exposure dose during the outdoor music festival was measured for each individual, and the dose of TWA was averaged 93.2 ± 2.39 dB(A), ranging from 87.9 to 97.7 dB(A). Our results of TWA were comparable with the noise exposure measurements at a Norwegian outdoor music festival (Tronstad and Gelderblom, 2016) and outdistanced the limit dose of daily noise exposure in most countries and the World Health Organization’s recommendations (Neitzel and Fligor, 2019). Since the effect on auditory function are associated with the duration and intensity of sound exposure (Peng et al., 2007; Le Prell et al., 2012), in this study, we did not detect any audiometric TTS, DPOAE amplitude decrement (a reflection of hair cell function), or alteration in MOC efferent nerve function or speech recognition ability amid noise for either males or females after outdoor festival noise exposure (see Figures 3–5 and Table 2). The only significant changes were the reversible decrement of ABR wave I amplitude at 1 day after exposure (see Figure 6).

The inconsistent effects on auditory function among studies with quantified exposure doses are probably due to the following reasons: (1) the different timepoints after noise exposure to follow-up auditory examinations, as Le Prell et al. (2012) showed that most TTSs recovered within 4 h after exposure; (2) the different doses of acute noise exposure between studies, since TTSs were detected in studies with higher noise exposure doses [approximately 100 dB(A) TWA] (Ramakers et al., 2016) but not with approximately 90 dB(A) TWA exposure (Grinn et al., 2017); (3) these data also provided important insight into the high variability across individuals in vulnerability to TTSs after music exposure. Thus, it is necessary to conduct more prospective studies with measurable exposure doses to determine which level of recreational noise exposure doses would induce the temporary or permanent auditory impairment.

### Optimal Metrics for the Assessment of Cochlear Synaptopathy and “Hidden Hearing Loss” in Humans

Numerous recent animal studies in mice (Kujawa and Liberman, 2009), rats (Lobarinas et al., 2017), guinea pigs (Shi et al., 2016), and rhesus monkeys (Valero et al., 2017) have demonstrated that moderate noise exposure that does not induce a PTS or hair cell death could result in cochlear synaptopathy, manifested as loss of a subset of synaptic connections between inner hair cells and afferent nerves and decreased ABR wave I amplitude in response to suprathreshold stimulus (Lobarinas et al., 2017), which is widely accepted as the primary cause of HHL. In humans, direct evidence of cochlear synaptopathy is based on extraction of the temporal bones (Viana et al., 2015; Wu et al., 2019); however, noise exposure history and auditory examination data are not always available for these tissues. To date, it is still unclear whether noise-induced cochlear synaptopathy occurs in humans and whether there are optimal audiological measurements to assess cochlear synaptopathy and HHL. According to previous studies, candidate metrics include ABR, the middle-ear muscle reflex (MEMR), envelope-following responses (EFR), and extended high-frequency (EHF) audiograms (Bramhall et al., 2019).

Most human studies used the amplitudes of ABR wave I or electrocochleogram peak I to indicate cochlear synaptopathy. Some previous studies on normal-hearing young veterans (Bramhall et al., 2017) and college music students (Liberman et al., 2016) have suggested that individuals with high doses of reported noise exposure may have a reduction in ABR wave I amplitude or ratio of the summating potential to the action potential; however, other recent studies did not find significant correlations between noise exposure history and electrophysiological metrics related to cochlear synaptopathy (Grinn et al., 2017; Prendergast et al., 2017a; Guest et al., 2018). Many factors may underlie the discordant conclusion of those studies, including the difficulty in quantification of self-reported lifetime noise exposure and the large individual variability of ABR wave I amplitudes in humans (Bharadwaj et al., 2019; Bramhall et al., 2019).

Benefiting from the perspective design of our study, we were able to perform a self-comparison by normalizing the post exposure ABR wave I amplitude to the baseline amplitude within each individual, as most animal studies did (Kujawa and Liberman, 2009). We were surprised to find a transient reduction in ABR wave I amplitude at 1 day after exposure with almost complete recovery at 14 days after exposure (see Figure 6 except for the right ear of male participants), which appears to be the temporary functional alteration of cochlear synapses and AN fibers, rather than the permanent loss of synaptic connections in previous animal studies. An explanation might be that humans are more resistant to noise-induced cochlear damage than experimental animals. Cochlear synaptopathy was observed in mice, rats, and guinea pigs at levels of approximately 100, 106, and 109 dB SPL octave band exposure for 2 h (Kujawa and Liberman, 2009; Shi et al., 2016; Lobarinas et al., 2017), while rhesus monkeys were more resistant to cochlear synaptopathy than rodents (Valero et al., 2017), resulting in predictions that the human ear is quite “robust” and resistant to damage from daily noise exposure. In contrast, another perspective study did not detect electrophysiological deficits at 1 day after acute recreational noise exposure (Grinn et al., 2017). The inconsistence might be due to the lower exposure doses [92.7 ± 7.7 dB(A) for 3.3 ± 0.9 h] in their study than those in our study (TWA range 93.2 ± 2.4 dB(A) for 7.34 ± 0.63 h).

In general, our results indicated that suprathreshold ABR wave I amplitude might be a proper auditory electrophysiology metric to detect the cochlear synaptic dysfunction. However, we failed to find a significant correlation between the sound exposure doses and any temporary or sustainable auditory function changes, although the ABR wave I amplitude alteration showed a trend of correlation with TWA without statistical significance (see Figures 7A,B). A potential explanation was the variable susceptibility of cochlear synaptopathy among individuals, since a previous study in mice suggested that decreasing sound levels by 3 dB can eliminate synaptic injury (Fernandez et al., 2015).

#### The Risk of Recreational Noise-Induced Cochlear Synaptopathy and Auditory Perceptual Disorder

Noise-induced cochlear synaptopathy was expected to induce not only neural deficits but also suprathreshold speech-processing abilities, especially in noisy environments (Skoe et al., 2019; Washnik et al., 2020). Previous animal studies indicated that AN fibers with lower spontaneous rates and higher response thresholds seemed to be more vulnerable to noise damage (Furman et al., 2013; Liberman and Kujawa, 2017). Thus, speech recognition in noise tests has been used to research cochlear synaptopathy in many human studies. Liberman et al. (2016) assessed the word recognition performance of 34 normal-hearing participants aged 18–41 years and found that participants with high risk of noise damage performed more poorly in the presence of ipsilateral noise. In contrast, a number of recent studies failed to reveal the significant association between noise exposure and auditory behavioral function in humans. A study including 138 normal-hearing participants aged 18–36 years reported little relation between lifetime noise exposure and a series of perceptual behavioral measures (Prendergast et al., 2017b). Several other studies reported no relation between noise exposure, ABR wave I amplitude, and speech recognition in noise in humans with clinically normal hearing (Grinn et al., 2017; Yeend et al., 2017; Guest et al., 2018). However, it is necessary to note that the different procedures of speech-in-noise tests used in various studies might have different degrees of listening task difficulty, which makes it complicated to compare performances in speech perception from one study to another.

In our study, we used the MHINT SNR changes after noise exposure to assess the alterations of speech recognition ability in noise for each individual. Although our results provide no evidence that acute noise exposure induces any speech perceptual deficit in noisy environments for normal-hearing young adults, we found that even minor alterations in speech recognition ability in noise were associated with transient ABR wave I amplitude changes after noise exposure (see Figures 7C,D). There might be some explanations for these results: (1) speech-in-noise performances could not directly represent the cochlear synaptic or AN function as the ABR wave I, (2) the speech-in-noise ability might be influenced by confounding central factors such as attention, working memory, and language in addition to peripheral effects (Yeend et al., 2017), and (3) the large individual variability of SNRs changes among subjects in this study. Thus, our findings indicated that the recreational noise-induced cochlear synaptic dysfunction could probably contribute to at least a bit of change in auditory perception ability in noisy background in humans.

### Strengths and Limitations

The main strength of this study was that prospective design and self-comparison make the data reliable. To our knowledge, this current study is one of the very few prospective studies that focused on the recreational noise-induced cochlear synaptopathy or HHL in humans. Another strength was the well-measured noise exposure level for each participant using the individual sound dosimeter in this study, which provides the accurate noise exposure doses. Moreover, we attempted to detect the effect on MOC efferent functional changes of acute recreational noise exposure, though no significance was found. Here we chose the CS on TEOAEs to evaluate the MOC efferent reflex because of its reliability (Stuart and Cobb, 2015). Since TEOAE is biased to low frequencies while DPOAE to high frequencies, DPOAE could be a choice in future noise-induced HHL studies. There are some other limitations. We did not perform the EHF audiograms in this study, which might be more sensitive to NIHL (Mehrparvar et al., 2011). We did not exclude participants with self-reported tinnitus in this study, while attenuated wave I amplitudes have been observed in normal human listeners with tinnitus compared with non-tinnitus controls (Schaette and McAlpine, 2011; Gu et al., 2012). The extent to which tinnitus is a symptomatic manifestation of noise-induced synaptopathy remains unclear.

In general, larger sample sizes and additional candidate cochlear synaptopathy-related metrics such as the compound action potential wave AP, ABR wave V amplitudes, EHF audiograms, EFRs, MEMR, and more auditory processing tests are needed to investigate the correlations in the future.

## Conclusion

Benefiting from the prospective design of this study, we were able to catch the transient ABR wave I amplitude decrement at 1 day post acute recreational noise exposure in normal hearing young adults. Our results indicated that the ABR wave I amplitude might be a sensitive metric to detect the noise-induced cochlear synaptopathy in humans, which also contributes to speech recognition ability in noise. Nevertheless, it should be noted that, although wave I of the ABR is the most direct non-invasive measure of cochlear synaptic and AN fidelity in humans, one of the obstacles for the use of the ABR to identify synaptopathy in humans is that wave I amplitude is highly variable across individuals. Overall, our study provides an insight into the potential recreational noise-induced cochlear synaptopathy and auditory speech perceptual difficulty in noisy backgrounds. With the increased prevalence of HHL, more attention should be paid to the prevention of recreational noise exposure-induced hearing impairment in humans.

## Data Availability Statement

The original contributions presented in the study are included in the article/Supplementary Material, further inquiries can be directed to the corresponding author/s.

## Ethics Statement

The studies involving human participants were reviewed and approved by the Ethics Committee of the Ninth People’s Hospital affiliated to Shanghai Jiao Tong University School of Medicine (SH9H-2019-T183-1). The patients/participants provided their written informed consent to participate in this study.

## Author Contributions

QW, ZH, and HW contributed to the conception and design of the study. LY, YH, and XW organized the database. QW and MQ performed the statistical analysis. QW and LY wrote the first draft of the manuscript. MQ and XW wrote sections of the manuscript. All authors contributed to manuscript revision, read, and approved the submitted version.

## Conflict of Interest

The authors declare that the research was conducted in the absence of any commercial or financial relationships that could be construed as a potential conflict of interest.
