# Supplementary material for: Acute Recreational Noise-Induced Cochlear Synaptic Dysfunction in Humans With Normal Hearing: A Prospective Cohort Study
Source: Front Neurosci. 2021 Apr 9;15:659011. doi: 10.3389/fnins.2021.659011 (PMC8062885; doi:10.3389/fnins.2021.659011)
Supplement: Supplementary file 1 [file Data_Sheet_1.docx]

Supplementary Material

# Supplementary Figures

**
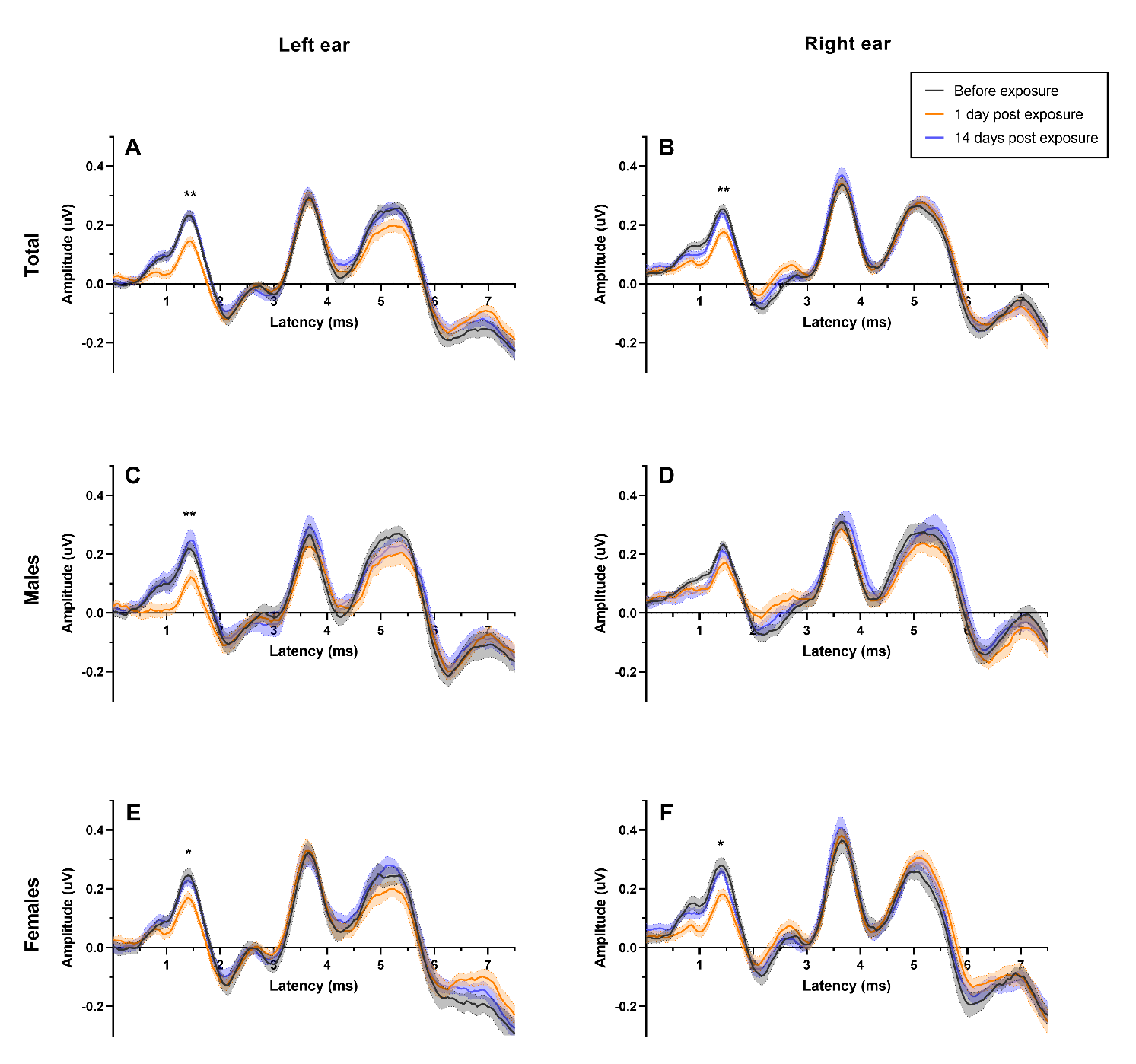
**

**Supplementary Figure 1.** **Effects of noise exposure on ABR wave forms.** The peak amplitude of wave I significantly decreased at one day post the outdoor music festival noise exposure (orange) and recovered to the baseline level (black) at 14 days post exposure (blue) for left ear and right ear of total participants (A, B) and females (E, F), as well as left ear (C) of males, but not right ear of which (D). Analyses were performed by the two-way repeated measures ANOVA. **P*<0.05, ***P*<0.01.


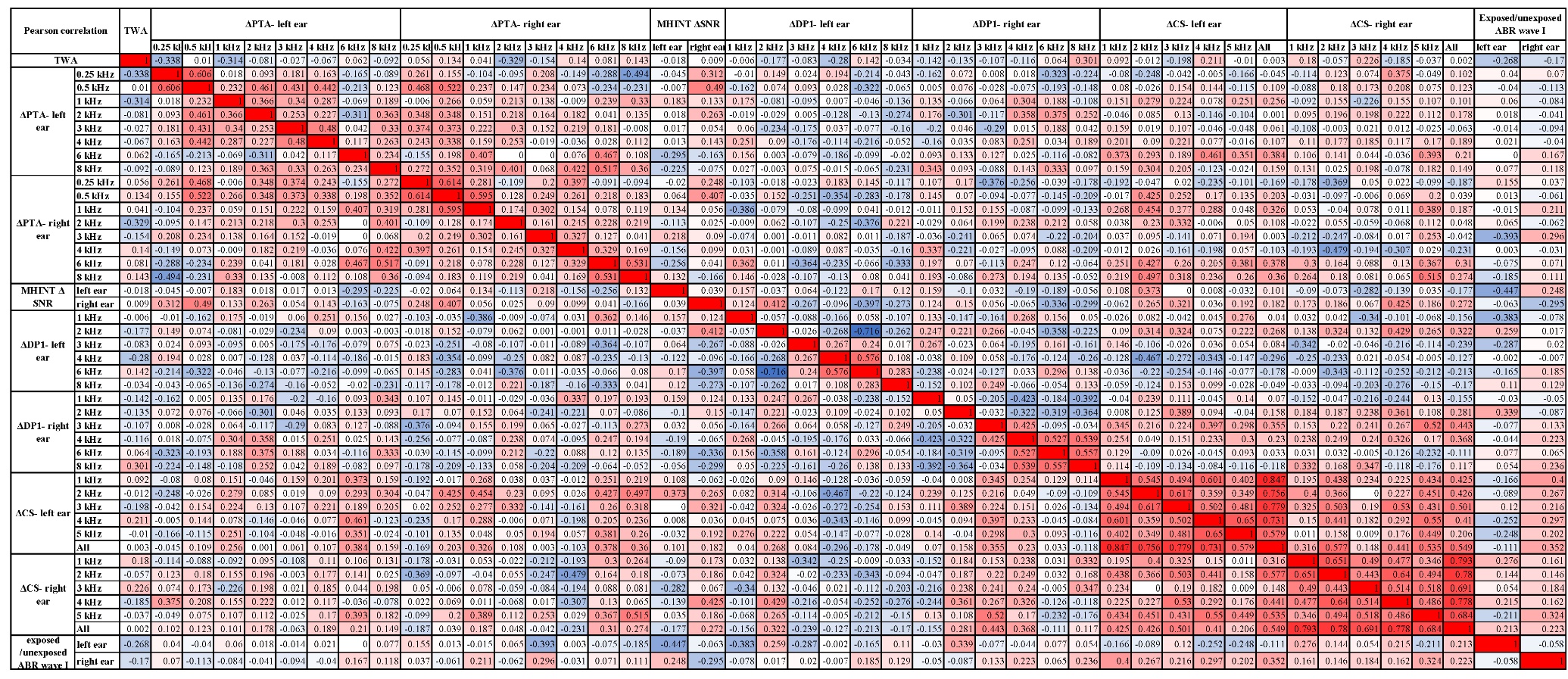


**Supplementary Figure 2.** **Pearson correlation analysis of TWA and auditory function changes at one day post exposure, we performed.** Pairwise correlation coefficients of TWA, changes on PTA thresholds, MHINT SNRs, DP1 amplitudes, CS on TEOAEs, and exposed/unexposed ratios of ABR wave I amplitudes at one day post the outdoor music festival noise exposure show in the grid. The red filling represents the positive correlation, the blue filling represents the negative correlation, the depth of shade of the color represents the degree of correlation. No significant correlation between the TWA and auditory function changes. Analyses were performed by the 2-tailed Pearson correlation analysis.
